# Estimating the Public Health and Economic Impact of Annual mRNA COVID-19 Vaccination for Adults Aged 50 and Older in South Korea’s Endemic Era

**DOI:** 10.3390/vaccines13040386

**Published:** 2025-04-03

**Authors:** Jaehee Jung, Dain Lee, Hee-Do Yang, Ah-Young Kim, Haeun Lee, Minkyoung Kang, Ekkehard Beck, Keya Joshi, Youngju Kang, Hye-Young Kang

**Affiliations:** 1 Yonsei Institute of Pharmaceutical Science, College of Pharmacy, Yonsei University, Incheon 21983, Republic of Korea; erica1127@yonsei.ac.kr (J.J.); dain.lee@yonsei.ac.kr (D.L.); minkyoung.kang@yonsei.ac.kr (M.K.); 2 Graduate Program of Industrial Pharmaceutical Science, College of Pharmacy, Yonsei University, Incheon 21983, Republic of Korea; heedo.yang@yonsei.ac.kr (H.-D.Y.); haeun1024@naver.com (H.L.); 3 College of Pharmacy, Ewha Womans University, Seoul 03760, Republic of Korea; pharmay@yonsei.ac.kr; 4 Moderna, Inc., 325 Binney St, Cambridge, MA 02142, USA; ekkehard.beck@modernatx.com (E.B.); keya.joshi@modernatx.com (K.J.); 5 Moderna Korea, Seoul 03185, Republic of Korea; youngju.kang@modernatx.com

**Keywords:** annual vaccination, costs, COVID-19, hospitalization, prevention, vaccine effectiveness, vaccine uptake

## Abstract

**Background/Objectives:** COVID-19 continues to challenge public health due to emerging variants. To mitigate this, the Korea Disease Control and Prevention Agency (KDCA) recommends annual COVID-19 vaccination, but uptake remains suboptimal. This study evaluates the public health and economic impact of annual mRNA COVID-19 vaccination for adults aged 50 and older in South Korea during the 2024–2025 season, focusing on hospitalizations and costs. **Methods:** We estimated hospitalizations prevented by the mRNA-1273 XBB.1.5 containing vaccine by calculating symptomatic infection incidence rates, hospitalization rates among unvaccinated individuals, vaccine effectiveness (VE) against hospitalization, and vaccination rates. Incidence rates among the unvaccinated with an annual vaccine were derived by adjusting overall infection rates based on vaccination coverage and VE against COVID-19 hospitalization rates. Hospitalization costs were obtained from a real-world dataset, integrating the KDCA’s COVID-19 confirmed cases with National Health Insurance claims data. Comparative analyses between mRNA-1273 and BNT162b2 used published meta-analysis results. **Results:** Assuming vaccination rates remain consistent with the 2023–2024 season, mRNA-1273 is projected to prevent 37,200 hospitalizations and save USD 77.2 million in healthcare costs during the 2024–2025 season compared to no annual vaccination. Compared to BNT162b2, it is expected to prevent an additional 13,260 hospitalizations saving USD 27.5 million. If vaccination rates increased to match influenza, hospitalizations prevented by mRNA-1273 could rise to 79,800 with USD 164.2 million in healthcare savings compared to no annual vaccination. **Conclusion:** Annual mRNA COVID-19 vaccination with mRNA-1273 substantially reduces hospitalizations and healthcare costs. Increasing vaccination rates are essential to maximize public health benefits.

## 1. Background

COVID-19 continues to pose a significant public health burden due to the emergence of new variants. In response to this, the Korea Disease Control and Prevention Agency (KDCA) recommends annual vaccination of COVID-19 vaccines for high-risk populations. For the 2024–2025 season, COVID-19 vaccine is administered from 11 October 2024 to 30 April 2025, with the goal of preventing infection, severe illness, and death among high-risk groups, including seniors aged 65 and older, immunocompromised individuals aged six months and older, and residents or patients in infection-prone facilities [1]. However, the vaccination rates during the 2023–2024 season were below expectations. Specifically, cumulative uptake of the mRNA COVID-19 vaccine (the vaccine type recommended by the KDCA for that season) from October 2023 to September 2024 reached only 19.62%, 44.03%, and 46.29% among individuals in the 60s, 70s, and 80+ age groups, respectively [2]. These rates are substantially lower than vaccination rates for the annual influenza vaccine during the 2022–2023 season, which were 59.10% for those in their 60s and 85.90% for individuals aged 70 and older [3]. This gap highlights the need to investigate barriers to COVID-19 vaccine uptake, such as insufficient awareness of risks associated with COVID-19, vaccine safety concerns, and financial constraints [4].

A recent real-world study in the United States found that adults aged 18 and older who received the XBB.1.5 mRNA COVID-19 vaccine (i.e., mRNA-1273) during the 2023–2024 season experienced a 33.1–38.7% reduction in risk for any medically attended COVID-19 event compared to unvaccinated individuals [5]. Furthermore, the vaccine showed a 58.7–61.1% effectiveness in preventing COVID-19-related hospitalizations [5]. Notably, the greater effectiveness observed in reducing hospitalizations suggests that annual vaccination could play a crucial role in preventing severe illness. Additionally, the protective effects were consistent across age groups: 35.3% effectiveness against medically attended COVID-19 for individuals aged 50 and older, compared to 38.7% for those aged 65 and above, and 61.1% effectiveness in preventing hospitalizations for individuals aged 50 and older, compared to 60.5% for those aged 65 and above [5]. These vaccine effectiveness estimates are consistent with previously published studies on XBB1.5 vaccine effectiveness [6].

On 21 July 2022, the KDCA recommended a fourth dose of the COVID-19 vaccine for individuals in their 50 s, citing their higher severity and fatality rates compared to those in their 40s, as well as the need to mitigate societal damage from the virus [7]. A survey by the Korean Ministry of Gender Equality and Family found that people in their 50s experienced the highest income losses due to COVID-19 [8], likely because this age group has the highest average income [9]. Similarly, Wang (2022) emphasized the global importance of protecting middle-aged workers from COVID-19 infection [10]. Given both the health risks and economic burdens faced by individuals aged 50 and older, extending the annual vaccination recommendation to individuals aged 50 and older may be beneficial.

Therefore, our study aims to quantify the potential public health benefits of annual COVID-19 vaccination by estimating the number of hospitalizations prevented among adults aged 50 and older in South Korea. We also evaluated the budgetary impact on the National Health Insurance (NHI) system and society resulting from these prevented events. Additionally, we estimated the enhanced public health and economic benefits if the uptake rate of COVID-19 vaccines reaches the level of influenza vaccinations. Finally, we compared the public health impact of two mRNA vaccines (mRNA-1273 vs. BNT162b2) using previously published comparative effectiveness data from a meta-analysis. This analysis estimating public health and economic benefits based on real-world evidence will help support increased vaccination uptake nationally.

## 2. Methods

We estimated the expected number of COVID-19 hospitalizations prevented by annual vaccination with the mRNA-1273 XBB.1.5 containing vaccine, by multiplying the population size of individuals aged 50 and older, the estimated COVID-19 symptomatic infection incidence rate among the unvaccinated, the COVID-19 hospitalization rates in the unvaccinated, the projected vaccination rate for 2024–2025 season, and the vaccine effectiveness (VE) against hospitalization. The expected savings of NHI spending resulting from the prevented hospitalization were computed by multiplying the expected number of COVID-19 hospitalizations prevented by the median cost for NHI-covered services in treating COVID-19 hospitalization. All costs are presented in US dollars (USD), applying a conversion rate of 1 USD = 1300 KRW.

Additionally, we compared the number of COVID-19 hospitalizations prevented and their associated costs between mRNA-1273 and BNT162b2, using published comparative effectiveness data. We best utilized published literature, government statistics, and secondary data available as data sources for each variable. In the following sections, we present data sources for each input variable and explain how the variables were operationalized.

### 2.1. COVID-19 Symptomatic Infection Incidence Rates Among the Unvaccinated

**Figure 1:**
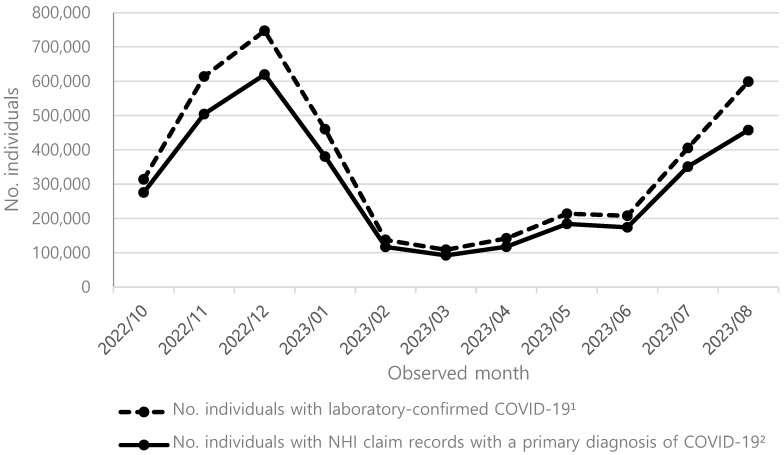
Laboratory-confirmed COVID-19 infection cases and healthcare utilization for COVID-19, October 2022–August 2023. Dotted line represents laboratory-confirmed COVID-19 cases, which include all individuals diagnosed with COVID-19 infection regardless of healthcare utilization. Solid line represents individuals with NHI claim records, which includes individuals who sought medical care for COVID-19 infection. NHI: National Health Insurance. 1. Data were provided by the Korea Disease Control and Prevention Agency, collected from October 2022 through August 2023. 2. Data source: Health Insurance Review Assessment Services Open Statistics during 2022–2023 season (i.e., October 2022 through August 2023). Diagnosis of COVID-19 was defined according to International Classification of Disease Code, 10th version (ICD-10 code) of U071 (coronavirus disease 2019, virus identified).

Since mandatory reporting of laboratory-confirmed COVID-19 cases has ceased as of 31 August 2023 in South Korea, the exact incidence rate at current is unavailable. Therefore, we estimated the ‘overall incidence rates for the 2024–2025 season’ based on the ratio of the number of patients utilizing healthcare services for COVID-19 treatments, according to the Health Insurance Review and Assessment Service (HIRA) Open Statistics from October 2022 to August 2023, to the number of laboratory-confirmed cases during the same period, provided by the KDCA (Figure 1). Healthcare services used for COVID-19 treatments were defined as NHI claims records with a primary diagnosis of COVID-19 (International Classification Disease code, 10th version [ICD-10 code]: U071 [COVID-2019, virus identified]). We estimated the overall incidence rates by multiplying the number of patients utilizing healthcare services for COVID-19 by the inverse of the ratio (Table 1). Then, the variable of ‘COVID-19 symptomatic infection incidence rates among the unvaccinated with an annual COVID-19 vaccine’ was estimated by adjusting the overall age-stratified incidence rate of COVID-19 infection by vaccine uptake rate and VE. This is carried out by applying VE of mRNA-1273 [5]. The specific estimation methods are presented in Appendix A.

### 2.2. COVID-19 Hospitalization Rates in the Unvaccinated

The age-specific likelihood of hospitalization with laboratory-confirmed symptomatic COVID-19 among unvaccinated individuals was derived from the KDCA COVID-19 National Health Insurance System (NHIS) cohort dataset, referred to as K-COV-N (Table 2). This dataset is specially developed by the government to enhance the real-world evidence regarding COVID-19. It integrates information from the KDCA’s COVID-19 confirmed case and vaccination databases with claims data from the NHIS. For this study, we used the K-COV-N dataset, which includes patients who used healthcare services with COVID-19 listed as the diagnosis (ICD-10 code U071) between 1 March 2022 and 28 February 2023. The study protocol was approved by the Institutional Review Board (IRB) of Yonsei University, Seoul, South Korea (IRB No. 7001988-202402-HR-2155-01E). Patient consent was waived due to the retrospective nature of the study and the robust personal information protection measures of the K-COV-N dataset. This dataset consists of de-identified claims records that obscure potentially identifiable information, including social security numbers, exact ages, and residential locations.

COVID-19 hospitalization data for unvaccinated individuals during this period were analyzed, with unvaccinated status defined as having no record of COVID-19 vaccination in the year preceding a COVID-19 diagnosis. Hospitalization rates were calculated by dividing the number of hospitalizations among unvaccinated individuals by the total number of confirmed cases in this group.

### 2.3. mRNA COVID-19 Vaccine Uptake Rate

It was assumed that the final vaccination rate for the mRNA COVID-19 annual vaccines in the 2024–2025 season would be the same as that for the 2023–2024 season (Table 2), which was obtained from the Infectious Disease Portal by KDCA [2].

### 2.4. Vaccine Effectiveness of mRNA-1273 Against COVID-19 Hospitalization

Given delays in obtaining real-world evidence for VE in South Korea, we used data from the United States to assess the effectiveness of the 2023–2024 Omicron XBB 1.5 containing mRNA-1273 in preventing COVID-19-related hospitalizations among adults aged 50 and older [5]. In this retrospective cohort study, 880,783 recipients of the mRNA-1273.815 vaccine, who were vaccinated between 12 September 2023 and 15 December 2023, were randomly 1:1 matched with individuals in the unvaccinated group. Both groups were monitored for COVID-19-related hospitalizations through 31 December 2023. A Cox regression model was used to estimate the hazard ratio for the outcome. As a result, the real-world effectiveness of the vaccine for adults aged 50 and older was estimated to be 61.1% (95% confidence interval [CI]: 54.3%–66.9%) [5].

For simplicity, the analysis considered only the cumulative one-year risk of COVID-19 hospitalization, assuming a consistent incidence rate across all months of the year, without accounting for potential fluctuations during periods of higher or lower incidence. Given that 99% of annual influenza vaccines in South Korea are administered within the first six weeks of the season [11], we estimated the average VE by assuming that everyone receives the COVID-19 annual vaccine at the start of the season, with its effectiveness experiencing a linear decline over time.

We, therefore, estimated the average VE over the year. More specifically, for VE against COVID-19 hospitalization, the average VE was calculated as (VE at month 1 + VE at month 12)/2, where VE at month 12 is calculated assuming waning occurs in each of the 11 months following the month when COVID-19 vaccination was given. The waning rates are based on a meta-analysis by Higdon et al. [12]. According to the Higdon study, an analysis of vaccine waning against the Omicron variant showed that after six months, protection against severe disease decreased by 8.2%. Based on these values, the average monthly waning effect was calculated as 1.37 percentage points (8.2%/6) for severe disease, and these values were used for our analysis.

At month 1, we calculated VE against hospitalization using the VE estimates provided by Kopel et al. [5] and back-calculated VE for month 2. Since Kopel et al. reported a median follow-up duration of 63 days (approximately two months), it was assumed that about two months of VE waning had already occurred. We assume that peak effectiveness occurs approximately two weeks after vaccination to account for immune lag [13,14]. We adjusted the VE at month 1 to account for this delay. Specifically, we assumed that the reported VE at month 1 reflects the midpoint of month 1, thereby incorporating 1.5 months of waning. Detailed calculation steps are outlined in Appendix B.

### 2.5. Comparative Effectiveness of mRNA-1273 over BNT162b2

The number of COVID-19 hospitalizations and its associated costs prevented by mRNA-1273 over BNT162b2 was determined using published comparative effectiveness data from a meta-analysis [15]. Twenty-four non-randomized real-world studies reporting COVID-19 outcomes in older adults aged 50 or above who received mRNA-1273 or BNT162b2 within the same study from March 2020 through June 2023 were included as being relevant for the meta-analysis. Based on the eight studies assessed in the meta-analysis, the study reported that the relative risk of hospitalization due to COVID-19 in individuals aged 50 or above vaccinated with mRNA-1273 over BNT162b2 was 0.65 (95% CI: 0.53–0.79). Using the same approach described in Section 2.4, we estimated the average annual VE for BNT162b2 (see Appendix C). We assume the same waning rate for BNT162b2 and mRNA-1273.

### 2.6. Median Cost of COVID-19 Hospitalization

Using K-COV-N data, we obtained median health care costs for NHI-covered services related to COVID-19 hospitalization with a primary diagnosis of COVID-19 between March 2022 and February 2023. Since the extent of health care resource consumption varies depending on the severity of hospitalization, we separately estimated median costs for each type of hospitalization: hospitalization using intensive care unit (ICU), hospitalization with ventilator, and general hospitalization neither using ICU nor ventilator. The weighted median costs for COVID-19 hospitalization were computed using proportion of each type as a weight (Table 3). Further, we included NHI-uncovered medical costs, transportation costs spent to visit healthcare institutions, and caregiver’s costs and productivity loss costs during hospitalization to estimate the costs from a societal perspective (Table 3). Vaccination costs, including both vaccine prices and administration expenses, were not considered in our study, as they are beyond the scope of its objectives.

### 2.7. Sensitivity Analysis

To assess the impact of uncertainties and assumptions regarding key parameters on the study results, we conducted sensitivity analyses (SAs). The parameters included in the SA were the VE of mRNA-1273 and the comparative VE of mRNA-1273 versus BNT162b2. The upper and lower bounds of the 95% confidence interval were used in the SA.

## 3. Results

The projected overall incidence rates of COVID-19 among adults aged 50 years and older in South Korea for the 2024–2025 season are detailed in Table 1. The incidence rate rises with age, starting at 10.68% for individuals aged 50–54 years and peaking at 16.45% for those aged 80 years and older. Based on NHI claims and COVID-19 healthcare service utilization rates, 2,793,500 infections are estimated in the 50+ age group, with 50% of infections occurring in adults 65 years and older.

The estimated number of COVID-19 hospitalizations prevented by annual vaccination with mRNA-based vaccines is provided in Table 2. Assuming vaccination rates for the 2023–2024 season are maintained, mRNA-1273 is estimated to prevent 34,400 hospitalizations in adults 65+ compared to no annual vaccination. Vaccinating adults 50+ could prevent almost 10% additional hospitalizations, totaling 37,200 hospitalizations averted compared to no annual vaccination. For the same season, BNT162b2 is projected to prevent 22,100 and 23,900 hospitalizations in adults 65+ and 50+, respectively, compared with no annual vaccination. The higher VE of mRNA-1273 accounts for its greater impact, particularly among older age groups. If vaccination rates were to reach levels comparable to influenza vaccination, hospitalizations prevented could increase to 79,800 for mRNA-1273.

Table 3 summarizes the economic burden of COVID-19 hospitalizations. The weighted median costs, considering all hospitalization types, showed a downward trend with age, decreasing from USD 3640 for individuals aged 55–59 to USD 3300 for those aged 80 and older from a societal perspective. Costs varied by hospitalization type, with ventilator use being the most expensive (USD 12,830 to USD 16,900), followed by ICU admissions (USD 7970 to USD 8250) and general hospitalizations (USD 2980 to USD 3420).

The estimated economic impact of hospitalizations prevented by annual COVID-19 vaccination is shown in Table 4. From the payer’s perspective, compared to no annual vaccination, mRNA-1273 is projected to avert USD 72 million in hospitalization costs in 65+ with an additional USD 5 million in savings when expanding vaccination to the 50+ population. Compared to no annual vaccination, BNT162b2 could avert USD 46.3 million and USD 49.7 million in hospitalization costs in the 65+ and 50+ populations, respectively, resulting in a difference of USD 28 million of potential healthcare costs averted in the 50+ population.

From the societal perspective, mRNA-1273 is expected to prevent USD 124.9 million in costs in the 50+ population compared to no annual vaccination, while BNT162b2 is estimated to prevent USD 80.4 million. Furthermore, if vaccination rates were to reach levels similar to those of influenza vaccination, the hospitalization costs avoided from a societal perspective could rise to USD 270.1 million for mRNA-1273.

The incremental cost difference between the two vaccines increases with age and with coverage, with the cost difference being most substantial in the oldest age group (80+) under influenza-like coverage. This highlights the greater impact in the group with the highest burden of severe disease.

Sensitivity analyses using the upper and lower bounds of the vaccine effectiveness for mRNA-1273 estimated that 32,400 to 37,400 hospitalizations could be prevented in the 50+ population during the 2024–2025 season compared to no annual vaccination. This corresponds to a potential prevention of treatment costs ranging from USD 108.9 million to USD 125.8 million from societal perspective. Using the 95% CI for the relative vaccine efficacy between mRNA-1273 and BNT162b2, mRNA-1273 was projected to prevent an additional 6000 to 6500 and 20,200 to 21,800 hospitalizations in the 65+ and 50+ populations, respectively. The associated incremental cost savings from hospitalization prevention ranged from USD 20.3 to USD 22.0 million and USD 67.6 to USD 73.4 million in the 65+ and 50+ populations, respectively. Full results are shown in Table 5.

## 4. Discussion

This study highlights the potential public health and economic benefits of annual mRNA COVID-19 vaccination among adults aged 65 years and older in South Korea, and shows the additional value of expanding vaccination to adults 50+, with particular focus on preventing severe disease and reducing hospitalization costs. Our estimates indicate that expanding vaccination from ages 65+ to 50+ would result in a 10% increase in prevented hospitalizations and associated cost savings. While this increase may seem modest, previous studies have shown that individuals in their 50s experienced the highest income and productivity losses [8] as well as significant societal impact due to COVID-19 [7]. Given this, our estimates—focused on healthcare utilization and treatment costs—likely capture only part of the broader consequences of the virus. Therefore, adopting a wider perspective beyond health care cost savings would provide a more comprehensive assessment of the potential value of expanding vaccination to adults 50+ or other vulnerable populations.

This study demonstrates that annual mRNA vaccination has the potential to prevent a substantial number of hospitalizations, reducing disease burden in the 50+ population. Assuming current vaccination rates, mRNA-1273 is projected to prevent approximately 37,200 hospitalizations, whereas BNT162b2, was estimated to prevent 23,900 hospitalizations (Table 2). If vaccine uptake improves to influenza vaccination levels, hospitalizations prevented could more than double for both vaccines, underscoring the critical role of increasing vaccination coverage to optimize public health outcomes. From an economic perspective, the prevention of hospitalizations translates into considerable treatment cost savings for both the NHI system and society. Compared to no annual vaccination, vaccination with mRNA-1273 in the 50+ population was associated with approximately USD 77.2 million in costs prevented from the payer’s perspective and USD 124.9 million from the societal perspective, compared to USD 49.7 million and USD 80.4 million, respectively, for BNT162b2 (Table 4). These findings align with previous real-world studies demonstrating the effectiveness of mRNA vaccines in preventing severe outcomes, particularly among older adults [16]. Based on the assumed higher VE of mRNA-1273 vs BNT162b2, we observe 12,300 to 13,300 additional hospitalizations averted in the 65+ and 50+ populations respectively, vaccinating with mRNA-1273 vs BNT162b2, suggesting that prioritizing vaccines with greater effectiveness could yield significant public health and economic advantages.

Despite these positive projections, low COVID-19 vaccination rates remain a major challenge. The gap between COVID-19 and influenza vaccination rates highlights the need for strategies to address vaccine hesitancy and logistical barriers. Based on evidence, enhanced public education campaigns may emphasize several key points: the serious health risks posed by COVID-19, the proven safety and effectiveness of mRNA vaccines, and the broader economic and societal benefits of widespread vaccination. Tailoring communication efforts to address specific concerns and misconceptions while making vaccination more accessible could significantly boost acceptance rates. Policies aimed at reducing financial barriers, such as subsidizing vaccine costs or incentivizing vaccinations, could also play a critical role. Co-administration of COVID-19 and influenza vaccines has gained attention as a strategy to improve vaccination rates against the concurrent circulation of both viruses [17]. Recent research indicates that giving these vaccines simultaneously across various population groups does not weaken the immune response [18,19,20]. Moreover, evidence suggests that co-administration may not increase safety risks compared to administering each vaccine separately [18,20].

This study has several limitations, which may lead to an over- or underestimation of our analysis. First, the projections rely on the assumption that incidence rates and healthcare utilization patterns observed in recent years will remain unchanged for the 2024–2025 season. However, these factors may shift as new variants emerge or the epidemiology of COVID-19 changes. Another key assumption of this study is that vaccination coverage will remain the same as in the previous season. This may not reflect future changes in public behavior or policy. Incorporating dynamic trends in vaccine uptake could improve the accuracy and relevance of future projections. Also, considering an alternative coverage scenario as a sensitivity analysis would be helpful. Additionally, we assume the incidence is constant across the one-year analysis period, which could lead to an underestimate of the public health burden. Second, the analysis does not account for the indirect benefits of vaccination, such as reduced transmission resulting from a herd immunity, which may underestimate the overall impact of annual vaccination [21].

Third, this study adopted a more conservative definition of COVID-19 cases. While previous studies broadly defined COVID-19 to include patients with both confirmed and unconfirmed viral infections, using ICD-10 codes, such as B342 (coronavirus infection, unspecified site), B972 (coronavirus as the cause of diseases classified elsewhere), U071 (coronavirus disease 2019, virus identified), and U072 (coronavirus disease 2019, virus not identified) [22], this study focused only on patients with clearly confirmed COVID-19 infection (ICD-10 code U071). If we had defined COVID-19 cases more broadly, the estimated hospitalization prevention benefits and cost reductions associated with annual COVID-19 vaccination would have been greater. Additionally, we only consider the cost savings due to hospitalizations and do not consider the impact of outpatient treatments which would result in an underestimation of healthcare costs due to COVID-19 infection.

Fourth, we used the most recent real-world evidence for the vaccine effectiveness (VE) of the mRNA-1273 vaccine and its comparative VE over BNT162b2. However, these values may change if new variants emerge or vaccine formulations are updated. To address this uncertainty, we conducted sensitivity analyses using the lower and upper bounds of the 95% confidence intervals for VE and comparative VE values, respectively, and presented the potential range of public health and economic impacts associated with annual COVID-19 vaccination. Across all scenarios, we found more hospitalizations averted using mRNA-1273 vs. BNT162b2 and associated healthcare cost savings.

Fifth, while the results were presented using 5-year age intervals to reflect age-specific differences, the main interpretation and discussion grouped individuals aged 50 and older as a single population. This approach aimed to evaluate the overall impact of expanding vaccine eligibility, but it may obscure differences in risk and resource use between younger and older subgroups. Future studies should consider subgroup-specific interpretations to better inform age-targeted vaccination strategies.

Finally, caution is warranted when interpreting our findings on the economic impact of annual vaccination. Our analysis focused solely on reductions in hospitalization costs, providing a limited estimate of economic impact, and did not account for net cost savings, which would consider both hospitalization and vaccination costs. Future studies should be conducted examining the net impact incorporating additional care settings to understand the full economic impact of COVID-19 vaccination in Korean adults.

## 5. Conclusions

In conclusion, annual mRNA COVID-19 vaccination among adults aged 50 and older in South Korea has the potential to prevent a substantial number of hospitalizations and significantly reduce COVID-19 treatment costs. Expanding vaccination eligibility from ages 65+ to 50+ could lead to a 10% increase in prevented hospitalizations and cost savings. Moreover, raising vaccination rates to levels seen with influenza vaccines could double these benefits, highlighting the urgent need for targeted interventions to improve vaccine uptake. These findings provide valuable evidence to guide public health policy and resource allocation for COVID-19 vaccination programs.

## Figures and Tables

**Table 1:** Projected age-specific overall incidence rate of COVID-19 for the 2024–2025 season in South Korea.

Age, Years	No. Population ^a^ (A)	No. Individuals with NHI Claim Records with a Primary Diagnosis of COVID-19 ^b^, (B)	% COVID-19 Patients Utilizing HCS ^c^, (C)	Estimated No. Individuals Infected with COVID-19 (D = BC)	Overall Incidence Rate, ^d^ % (E = D/A)
50–54	4,525,612	411,837	85.24	483,165	10.68
55–59	4,070,751	352,401	85.24	413,435	10.16
60–64	4,258,205	415,619	83.44	498,125	11.70
65–69	3,274,183	369,504	83.44	442,856	13.53
70–74	2,234,677	275,075	82.83	332,108	14.86
75–79	1,637,098	204,626	82.83	247,052	15.09
80+	2,289,858	283,133	75.15	376,751	16.45
Total	22,290,384			2,793,492	

NHI, National Health Insurance; HCS, healthcare service. ^a^ Data source: Korea Statistical Office, 2023. ^b^ Data source: Health Insurance Review and Assessment Services (HIRA) Open Statistics during 2022–2023 season (i.e., October 2022 through August 2023). Diagnosis of COVID-19 was defined according to the International Classification of Disease Code, 10th version (ICD-10 code) of U071 (coronavirus disease 2019, virus identified). ^c^ It is estimated using data provided by the Korea Disease Control and Prevention Agency and data by HIRA Open Statistics for October 2022 through August 2023. ^d^ Overall incidence refers to the incidence rate, regardless of vaccination status.

**Table 2:** Estimated number of COVID-19 hospitalizations prevented by annual vaccination in the 2024–2025 season in South Korea.

Age, Years	No. Population (A)	Incidence in UV, % (B)	COVID-19 Hospz. in UV, % (C)	VR (If Increase to VR of IV), % (D)	VE by mRNA-1273, % (E)	VE by BNT162b2, % (F)	No. Hospz. Prevented by mRNA-1273 ^a^	No. Hospz. Prevented by BNT162b2 ^b^	Difference in No. Hospz. Prevented: mRNA-1273 vs. BNT162b2
50–54	4,525,612	10.76	2.72	3.56 (35.10)	55.62	35.79	262 [2581]	168 [1660]	93 [920]
55–59	4,070,751	10.23	3.49	3.56 (35.10)	55.62	35.79	288 [2842]	185 [1828]	103 [1013]
60–64	4,258,205	12.21	3.93	19.62 (59.10)	55.62	35.79	2228 [6712]	1434 [4318]	795 [2394]
65–69	3,274,183	14.11	4.67	19.62 (59.10)	55.62	35.79	2353 [7087]	1514 [4560]	839 [2527]
70–74	2,234,677	16.39	6.09	44.03 (85.90)	55.62	35.79	5467 [10,665]	3517 [6862]	1949 [3803]
75–79	1,637,098	16.65	9.01	44.03 (85.90)	55.62	35.79	6015 [11,736]	3870 [7551]	2145 [4185]
80+	2,289,858	18.24	19.12	46.29 (85.90)	55.62	35.79	20,565 [38,163]	13,231 [24,554]	7334 [13,610]
Total	22,290,384						37,178 [79,785]	23,920 [51,332]	13,258 [28,453]

Hospz., hospitalization; IV, influenza vaccine; UV, unvaccinated individuals; VR, vaccinate rate. ^a^ Expected number of hospitalizations prevented by mRNA-1273 vaccine was estimated as the product of (A), (B), (C), (D), and (E). ^b^ Expected number of hospitalizations prevented by the BNT162b2 vaccine was estimated as the product of (A), (B), (C), (D), and (F). Values in [ ] refer to the estimated number of hospitalizations prevented if vaccination rate of COVID-19 vaccines increases to vaccination rate of influenza vaccines in the 2022–2023 season.

**Table 3:** Economic burden of treating hospitalized COVID-19 infections.

	Type of Hospitalization, %			Median Costs from Payer’s Perspective ^a^				Median Costs from Societal Perspective ^b^			
Age, Years	General	ICU	Ventilator Use	General	ICU	Ventilator Use	Weighted Median ^c^	General	ICU	Ventilator Use	Weighted Median ^c^
50–54	97.41	1.61	0.98	1646	5569	11,738	1808	3417	8206	16,879	3626
55–59	96.75	2.08	1.17	1646	5569	11,738	1845	3386	8170	16,813	3643
60–64	96.69	2.07	1.24	1646	5569	11,738	1852	3265	8032	16,194	3525
65–69	95.87	2.44	1.68	1828	5899	9818	2061	3199	8248	13,194	3490
70–74	95.21	2.88	1.68	1828	5899	9818	2097	3110	8134	13,048	3444
75–79	94.39	3.36	2.25	1828	5899	9818	2144	2978	7964	12,831	3367
80+	95.08	3.46	1.46	1828	5899	9818	2085	2978	7964	12,831	3294

ICU, intensive care unit. Costs are presented in US dollars at 2024 value (1 USD equals 1300 KRW). Data source: COVID-19 hospitalization cases from March 2022 through February 2023 identified from the Korea Disease Control and Prevention Agency (KDCA) COVID-19 National Health Insurance System (NHIS) cohort dataset. ^a^ Costs from payer’s perspective include National Health Insurance (NHI)-covered medical costs during hospitalization. ^b^ Costs from societal perspective include NHI-covered and non-NHI-covered medical costs, transportation costs for healthcare visits, caregiver’s costs, and productivity loss costs during hospitalization. ^c^ Weighted median costs were calculated using proportion of each type of hospitalization (i.e., general, ICU, and ventilator use hospitalization) as a weight.

**Table 4:** Estimated costs of COVID-19 hospitalizations prevented by annual vaccination in the 2024–2025 season in South Korea.

	Payer’s Perspective ^a^			Societal Perspective ^b^		
Age, Years	Costs of Hospz. Prevented by mRNA-1273	Costs of Hospz. Prevented by BNT162b2	Difference: mRNA-1273 vs. BNT162b2	Costs of Hospz. Prevented by mRNA-1273	Costs of Hospz. Prevented by BNT162b2	Difference: mRNA-1273 vs. BNT162b2
50–54	473,138 [4,762,543]	304,409 [3,001,340]	168,729 [1,761,204]	949,066 [9,357,363]	610,614 [6,020,375]	338,452 [3,336,988]
55–59	531,875 [5,244,048]	342,200 [3,373,935]	189,675 [1,870,113]	1,049,921 [10,351,751]	675,502 [6,660,148]	374,419 [3,691,603]
60–64	4,127,709 [12,433,618]	2,655,700 [7,999,587]	1,472,008 [4,434,031]	7,853,485 [23,656,521]	5,052,804 [15,220,220]	2,800,681 [8,436,302]
65–69	4,850,015 [14,609,372]	3,120,420 [9,399,432]	1,729,595 [5,209,941]	8,211,422 [24,734,711]	5,283,095 [15,913,909]	2,928,327 [8,820,802]
70–74	11,465,281 [22,368,104]	7,376,574 [14,391,273]	4,088,706 [7,976,831]	18,829,227 [36,734,741]	12,114,418 [23,634,533]	6,714,810 [13,100,208]
75–79	12,896,622 [25,160,568]	8,297,476 [16,187,899]	4,599,146 [8,972,669]	20,253,431 [39,513,281]	13,030,727 [25,422,200]	7,222,704 [14,091,081]
80+	42,883,131 [79,577,899]	27,590,306 [51,199,121]	15,292,825 [28,378,778]	67,752,428 [125,727,664]	43,590,806 [80,891,126]	24,161,622 [44,836,537]
Total	77,227,770 [164,156,153]	49,687,087 [105,552,586]	27,540,684 [58,603,567]	124,898,980 [270,076,031]	80,357,965 [173,762,510]	44,541,015 [96,313,522]

All costs are presented in US dollars (1 USD equals 1300 KRW). Costs in [ ] refer to the estimated costs prevented if vaccination rate of COVID-19 vaccines increase to vaccination rate of influenza vaccines in 2022–2023 season. ^a^ Costs from payer’s perspective include National Health Insurance (NHI)-covered medical costs during hospitalization. ^b^ Costs from societal perspective include NHI-covered and non-NHI-covered medical costs, transportation costs for healthcare visits, caregiver’s costs, and productivity loss (PL) costs during hospitalization.

**Table 5:** Sensitivity analysis results for variations in the vaccine effectiveness of mRNA-1273.815 and its comparative effectiveness versus BNT162b2.

Age, Years	Applying 95% CI of VE of mRNA-1273				Applying 95% CI of rVE of mRNA-1273 vs. BNT162b2			
	Lower Bond		Upper Bound		Lower Bound		Upper Bound	
	No. Hospz. Prevented by mRNA-1273	Hospz. Costs Prevented by mRNA-1273 (Societal Perspective)	No. Hospz. Prevented by mRNA-1273	Hospz. Costs Prevented by mRNA-1273 (Societal Perspective)	Difference in No. Hospz. Prevented: mRNA-1273 vs. BNT162b2	Difference in Hospz. Costs Prevented: mRNA-1273 vs. BNT162b2 (Societal Perspective)	Difference in No. Hospz. Prevented: mRNA-1273 vs. BNT162b2	Difference in Hospz. Costs Prevented: mRNA-1273 vs. BNT162b2 (Societal Perspective)
50–54	230 [2264]	832,572 [8,208,785]	287 [2828]	1,040,119 [10,255,108]	46 [454]	167,123 [1,647,757]	154 [1516]	557,546 [5,497,156]
55–59	253 [2493]	921,048 [9,081,116]	316 [3114]	1,150,651 [11,344,897]	51 [500]	184,883 [1,822,861]	169 [1669]	616,796 [6,081,328]
60–64	1950 [5873]	6,871,528 [20,698,638]	2358 [7103]	8,311,501 [25,036,173]	392 [1182]	1,382,936 [4,165,726]	1309 [3943]	4,613,675 [13,897,462]
65–69	2059 [6201]	7,184,710 [21,642,016]	2490 [7501]	8,690,313 [26,177,242]	414 [1248]	1,445,966 [4,355,586]	1382 [4164]	4,823,952 [14,530,864]
70–74	4762 [9291]	16,403,718 [32,002,710]	5473 [10,677]	18,850,709 [36,776,651]	963 [1878]	3,315,677 [6,468,696]	3211 [6265]	11,061,579 [21,580,504]
75–79	5240 [10,224]	17,644,461 [34,423,329]	6022 [11,749]	20,276,538 [39,558,360]	1059 [2067]	3,566,468 [6,957,975]	3534 [6894]	11,898,254 [23,212,809]
80+	17,909 [33,233]	58,999,726 [109,485,342]	20,480 [38,005]	67,471,048 [125,205,509]	3621 [6720]	11,930,665 [22,139,644]	12,082 [22,420]	39,802,420 [73,861,047]
Total	32,402 [69,579]	108,857,762 [235,541,937]	37,426 [80,977]	125,790,879 [274,353,940]	6547 [14,050]	21,993,719 [47,558,245]	21,841 [46,871]	73,374,221 [158,661,171]

CI, confidence interval; Hospz., hospitalization; rVE, comparative effectiveness; VE, vaccine effectiveness.

## Data Availability

The primary data used in this study is the Korea Disease Control and Prevention Agency (KDCA) COVID-19 National Health Insurance System (NHIS) cohort dataset, a publicly accessible resource. Access requires submitting a study proposal and an Institutional Review Board (IRB) approval statement via https://nhiss.nhis.or.kr. Additional data used in this study can be obtained through the references cited in the manuscript.

## Appendix A. Calculation Steps for COVID-19 Symptomatic Infection Incidence Rate Among the Unvaccinated

Incidence rate *in the vaccinated* = Incidence rate in the unvaccinated × (1 − VE against infection)

*Overall* incidence rate = Incidence rate in the vaccinated × % vaccinated

+ Incidence rate in the unvaccinated × (1 − % vaccinated)

= Incidence rate in the unvaccinated × (1 − VE against infection) × % vaccinated

+ Incidence rate in the unvaccinated × (1 − % vaccinated)

Incidence rate in the unvaccinated

= Overall incidence rate/{(1 − VE against infection) × % vaccinated + (1 − % vaccinated)}

## Appendix B. Calculation Steps for Average Annual Vaccine Effectiveness of mRNA-1273 Vaccine

Average yearly VE of mRNA-1273.815 = (VE at 1st month + VE at 12th month)/2

={(VE + 1.5 × monthly waning %) + (VE at 1st month − monthly waning% × 11)}/2

= [(61.10% + 1.5 × 1.37%) + {(61.10% + 1.5 × 1.37%) − 1.37% × 11}]/2

= (63.16% + 48.09%)/2 = 55.62%

## Appendix C. Calculation Steps for Average Annual Vaccine Effectiveness Rates of BNT161b2 Vaccine

Risk = 1 − VE

Relative VE of mRNA-1273 vs. BNT162b2 = 1 − Risk Ratio of mRNA-1273 vs. BNT162b2

Risk Ratio of mRNA-1273 vs. BNT162b2 = 1 − Relative VE of mRNA-1273 vs. BNT162b2

Risk of mRNA-1273/Risk of BNT161b2 = (1 − VE of mRNA-1273)/(1 − VE of BNT162b2)

(1 − VE of BNT162b2) = (1 − VE of mRNA-1273)/Risk Ratio of mRNA-1273 vs. BNT162b2

VE of BNT162b2 = 1 − (1 − VE of mRNA-1273)/Relative VE of mRNA-1273 vs. BNT162b2

= 1 − (1 − 63.16%)/0.65

= 43.32%

Average yearly VE of BNT162b2 = (VE at 1st month + VE at 12th month)/2

= VE at 1st month + (VE at 1st month − monthly waning% × 11)}/2

= {43.32% + (43.32% − 1.37% × 11)}/2

= (43.32% + 28.25%)/2 = 35.79%

## References

[1] Korea Disease Control and Prevention Agency COVID-19 Vaccination Starts from October 11. https://www.kdca.go.kr/board/board.es?mid=a20501010000&bid=0015&list_no=726216&cg_code=&act=view&nPage=4&newsField=.

[2] Korea Disease Control and Prevention Agency COVID-19 Vaccination. https://dportal.kdca.go.kr/pot/www/CVID19/PRVNTN/VCNTN.jsp?seq=TAB1_1.

[3] Korean Statistical Information Service Trend of Influenza Vaccination Rate. https://kosis.kr/statHtml/statHtml.do?sso=ok&returnurl=https%3A%2F%2Fkosis.kr%3A443%2FstatHtml%2FstatHtml.do%3Flist_id%3D117_11702_A01_077%26obj_var_id%3D%26seqNo%3D%26tblId%3DDT_11702_N083%26vw_cd%3DMT_OTITLE%26orgId%3D177%26path%3D%252Fcommon%252Fmeta_.

[4] Hong S., Son Y., Lee M., Lee J.H., Park J., Lee H., Dragioti E., Fond G., Boyer L., López Sánchez G.F. (2024). Association between sociodemographic factors and vaccine acceptance for influenza and SARS-CoV-2 in South Korea: Nationwide Cross-Sectional Study. JMIR Public Health Surveill..

[5] Kopel H., Araujo A.B., Bogdanov A., Zeng N., Winer I., Winer-Jones J.P., Lu T., Marks M.A., Bonafede M., Nguyen V.H. (2024). Effectiveness of the 2023–2024 Omicron XBB. 1.5-containing mRNA COVID-19 vaccine (mRNA-1273.815) in preventing COVID-19–related hospitalizations and medical encounters among adults in the United States. Open Forum Infect. Dis..

[6] Ma K.C., Surie D., Lauring A.S., Martin E.T., Leis A.M., Papalambros L., Gaglani M., Columbus C., Gottlieb R.L., Ghamande S. (2024). Effectiveness of updated 2023–2024 (Monovalent XBB.1.5) COVID-19 vaccination against SARS-CoV-2 Omicron XBB and BA.2.86/JN.1 lineage hospitalization and a comparison of clinical severity-IVY Network, 26 hospitals, October 18, 2023-March 9, 2024. Clin. Infect. Dis..

[7] Korea Disease Control and Prevention Agency Announcement of the Status and Schedule of Large-Scale COVID-19 Seroprevalence Survey. https://www.kdca.go.kr/board/board.es?mid=a20501010000&bid=0015&list_no=720181&cg_code=&act=view&nPage=3&newsField=202207.

[8] Korea Statistical Information Service Life Changes Caused by COVID-19. https://kosis.kr/statHtml/statHtml.do?sso=ok&returnurl=https%3A%2F%2Fkosis.kr%3A443%2FstatHtml%2FstatHtml.do%3Fconn_path%3DMT_ZTITLE%26list_id%3DB_13_001%26obj_var_id%3D%26seqNo%3D%26tblId%3DDT_154021_22AA006300%26vw_cd%3DMT_ZTITLE%26itm_id%3D%26language%3Dkor%26lang_mode%3Dko%26orgId%3D154%26.

[9] Korea Statistical Information Service Status of Subscribers by Standard Monthly Income Bracket and Age Group. https://kosis.kr/statHtml/statHtml.do?sso=ok&returnurl=https%3A%2F%2Fkosis.kr%3A443%2FstatHtml%2FstatHtml.do%3Fconn_path%3DMT_ZTITLE%26list_id%3D322_32202_02%26obj_var_id%3D%26seqNo%3D%26tblId%3DDT_32202_B019_1%26vw_cd%3DMT_ZTITLE%26itm_id%3D%26language%3Dkor%26lang_mode%3Dko%26orgId%3D322%26.

[10] Wang F., Wang J.-D. (2022). Estimating US earnings loss associated with COVID-19 based on human capital calculation. Int. J. Environ. Res. Public Health.

[11] Jang E.J., Lee H.R., Choi Y.H., Kim G. (2019). Immunization program against influenza in Korea; The 2018–2019 influenza season. Public Health Wkly. Rep..

[12] Higdon M.M., Baidya A., Walter K.K., Patel M.K., Issa H., Espié E., Feikin D.R., Knoll M.D. (2022). Duration of effectiveness of vaccination against COVID-19 caused by the omicron variant. Lancet Infect. Dis..

[13] Danza P., Koo T.H., Haddix M., Fisher R., Traub E., OYong K., Balter S. (2022). SARS-CoV-2 infection and hospitalization among adults aged >/=18 years, by vaccination status, before and during SARS-CoV-2 B.1.1.529 (Omicron) variant predominance—Los Angeles County, California, November 7, 2021-January 8, 2022. MMWR Morb. Mortal. Wkly. Rep..

[14] Tenforde M.W., Olson S.M., Self W.H., Talbot H.K., Lindsell C.J., Steingrub J.S., Shapiro N.I., Ginde A.A., Douin D.J., Prekker M.E. (2021). Effectiveness of Pfizer-BioNTech and Moderna vaccines against COVID-19 among hospitalized adults aged >/=65 Years—United States, January-March 2021. MMWR Morb. Mortal. Wkly. Rep..

[15] Kavikondala S., Haeussler K., Wang X., Bausch-Jurken M.T., Nassim M., Mishra N.K., Malmenäs M., Sharma P., Van de Velde N., Green N. (2024). Comparative effectiveness of mRNA-1273 and BNT162b2 COVID-19 vaccines among older adults: Systematic literature review and meta-analysis using the GRADE Framework. Infect. Dis. Ther..

[16] Xu K., Wang Z., Qin M., Gao Y., Luo N., Xie W., Zou Y., Wang J., Ma X. (2023). A systematic review and meta-analysis of the effectiveness and safety of COVID-19 vaccination in older adults. Front. Immunol..

[17] Kwon S.L., Kim S.-Y., Song M., Lee H.-M., Ban S.-H., Lee M.-S., Jeong H. (2024). Assessing the determinants of influenza and COVID-19 vaccine co-administration decisions in the elderly. Hum. Vaccin. Immunother..

[18] Murdoch L., Quan K., Baber J.A., Ho A.W., Zhang Y., Xu X., Lu C., Cooper D., Koury K., Lockhart S.P. (2023). Safety and immunogenicity of the BNT162b2 vaccine co-administered with seasonal inactivated influenza vaccine in adults. Infect. Dis. Ther..

[19] Xie Y., Tian X., Zhang X., Yao H., Wu N. (2023). Immune interference in effectiveness of influenza and COVID-19 vaccination. Front. Immunol..

[20] Shenyu W., Xiaoqian D., Bo C., Xuan D., Zeng W., Hangjie Z., Qianhui Z., Zhenzhen L., Chuanfu Y., Juan Y. (2022). Immunogenicity and safety of a SARS-CoV-2 inactivated vaccine (CoronaVac) co-administered with an inactivated quadrivalent influenza vaccine: A randomized, open-label, controlled study in healthy adults aged 18 to 59 years in China. Vaccine.

[21] Wang J., Chan Y.-C., Niu R., Wong E.W., van Wyk M.A. (2022). Modeling the impact of vaccination on COVID-19 and its Delta and Omicron variants. Viruses.

[22] Choi Y., Kang M., Shin D.H., Jung J., Choi S.J., Kim N.-H., Moon S.M., Song K.-H., Kim E.S., Jung J. (2023). Antibiotic prescription in patients with coronavirus disease 2019: Analysis of national health insurance system data in the Republic of Korea. J. Korean Med. Sci..

